# Vitamin E (α-tocopherol) supplementation in diabetic rats: effects on the proximal colon

**DOI:** 10.1186/1471-230X-9-88

**Published:** 2009-11-23

**Authors:** Luciana P Roldi, Renata VF Pereira, Eleandro A Tronchini, Gabriela V Rizo, Célia R Scoaris, Jacqueline N Zanoni, Maria RM Natali

**Affiliations:** 1 Department of Morphophysiological Sciences, Laboratory of Enteric Neurons, State University of Maringá, Brazil

## Abstract

**Background:**

Neuropathy is one of the complications caused by diabetes mellitus which is directly related to the gastrointestinal manifestations of the disease. Antioxidant substances, such as vitamin E, may play an important role in the reduction of the neurological damage caused by diabetes mellitus. The aim of the present study was to determine whether vitamin E (α-tocopherol) at different concentrations induces any effects on the morphology of the intestinal wall and intrinsic innervation in the proximal colon of diabetic rats.

**Methods:**

Thirty rats (90-day-old) were assigned to the following groups: N (normoglycemic), NE1 (normoglycemic supplemented with vitamin E 0.1%), NE2 (normoglycemic supplemented with vitamin E 2%), D (diabetic), DE1 (diabetic supplemented with vitamin E 0.1%), and DE2 (diabetic supplemented with vitamin E 2%). Animals received vitamin E supplementation for 120 days and were sacrificed when they were 210 days old. The proximal colon of each animal was subjected to histology to study the intestinal wall and goblet cells and processed for whole-mount preparations to morphoquantitatively determine the total myenteric population.

**Results:**

Supplementation with vitamin E significantly reduced glycemia and glycated hemoglobin values and preserved the number of myenteric neurons in group DE2, without affecting intestinal area or thickness of the intestinal wall or muscular tunic.

**Conclusion:**

Vitamin E (2%) influenced the glycemic parameters and had a neuroprotective effect on the total myenteric population, but the morphometric characteristics of the intestinal wall were unaffected.

## Background

Diabetes mellitus causes imbalances and pathological changes in several tissues. All types of diabetes mellitus are characterized by hyperglycemia and the development of specific microvascular pathologies, including in retina (retinopathy), renal glomeruli (nephropathy), and peripheral nerves (neuropathy), the last of which is most common in diabetic patients [1].

The manifestations of diabetic neuropathy lead to many symptoms in the gastrointestinal tract, including diabetic gastroparesy, diabetic enteropathy, esophageal motor dysfunction, colonic hypomobility, and rectum-anal dysfunction [2]. Morphological consequences of diabetic neuropathy, such as increased total area of the intestinal wall in the small intestine, hypertrophy [3] and hyperplasia of the intestinal mucosa [4], and an increased number of goblet cells [5], are also observed.

Vinik [6] found that diabetes-induced neuropathy in the gastrointestinal tract is related to alterations in myenteric neurons in the enteric nervous system that are present in the tubular wall of the digestive tract [7]. Recent studies have shown severe alterations of the enteric nervous system in rat models of experimental diabetes, such as a reduction in enteric neurons in the stomach [8], duodenum [9], ileum [10,11], colon [12], cecum [13], and proximal colon [14], and alterations in the area of neuronal cellular bodies in the ileum [10,11], colon [12], and cecum [13]. Hyperglycemia has been shown to be responsible for the development and progression of neuropathy, promoting loss of function and decreasing neuronal survival [15] due to alterations in blood flow, increases in vascular permeability, and decreases in neuronal trophic factors [16]. Accumulation of final glycation products (AGEs), polyol pathway hyperactivity, and an increase in oxidative stress are among the described mechanisms explaining how hyperglycemia damages the nervous system.

Diabetes-induced oxidative stress generates reactive oxygen species and an imbalance among antioxidants. Bhor et al. [17] reported altered activity in primary antioxidant enzymes (e.g., catalase, superoxide dismutase, and glutathione peroxidase) and an increase in lipid peroxidation and carbonyl protein content, thus ensuring the occurrence of oxidative stress in diabetic rats.

Several antioxidant enzymatic and non-enzymatic systems in the cell inactivate free radicals to reduce the damage caused by them. These antioxidants include glutathione, enzymatic systems, and vitamins A, C, and E [18]. The effect of vitamin E on free radicals is mainly important for preventing or delaying many degenerative diseases, such as cancer, cardiovascular inflammatory diseases, cellular alterations attributable to the aging process, and neurological diseases [19]. Vitamin E has eight different natural forms: α-, β-, δ-, and γ-tocopherols and α-, β-, δ-, and γ-tocotrienols. α-Tocopherol constitutes the most biologically active form [20].

In diabetic patients, Reunanen et al. [21] verified that vitamin E promoted a reduction in the indicators of oxidative stress and protein glycation. Prior studies in diabetic rats that received vitamin E treatment found a reduction in lipid peroxidation, an increase in superoxide dismutase activity [22], an increase in nervous system conductance velocity [23,24], and protection against nervous system dysfunction [25,26].

The goal of the present study was to determine whether vitamin E (α-tocopherol) at two different concentrations has any influence on intestinal wall morphology and myenteric neurons in the proximal colon of diabetic rats.

## Methods

### Animal procedure

The present study used 30 albino male adult Wistar rats (*Rattus norvegicus*) from the Central Biotery of the State University of Maringa (UEM). All procedures described in this study were approved by the Ethics Committee on Animal Experimentation of UEM. When rats were 90 days old, they were kept in polypropylene boxes for 120 days in the biotery under controlled temperature (24 ± 2°C) and illumination (12 h/12 h light/dark cycle) with *ad libitum *access to water and food. Animals from the normoglycemic (N) and diabetic (D) groups received padronized Nuvital^® ^chow pellets (Nuvilab, Colombo, PR, Brazil). The groups allocated to groups NE1 and NE2 (normoglycemic supplemented with vitamin E 0.1% and 2%, respectively) and groups DE1 and DE2 (diabetics supplemented with vitamin E 0.1% and 2%, respectively) received vitamin E (α-tocopherol; Zhejiang NHU, China) supplementation added to the padronized chow at concentrations of 0.1% [23] and 2%.

Animals were allocated to one of six groups (*n *= 5/group): N (normoglycemic rats receiving padronized chow), NE1 (normoglycemic rats receiving padronized chow supplemented with vitamin E 0.1%), NE2 (normoglycemic rats receiving padronized chow supplemented with vitamin E 2%), D (diabetic rats receiving padronized chow), DE1 (diabetic rats receiving padronized chow supplemented with vitamin E 0.1%), and DE2 (diabetic rats receiving padronized chow supplemented with vitamin E 2%).

To induce diabetes, after prior fasting for 14 h, animals from groups D, DE1, and DE2 were injected (into the penis vein) with streptozotocin (Sigma, St. Louis, MO, USA) at a concentration of 35 mg/kg body weight [27] dissolved in 10 mM citrate buffer, pH 4.5.

Glucose levels after diabetes induction were determined by the glucose oxidase method [28] to confirm induction of the disease. Only animals with >250 mg/dl glycemia were retained in groups D, DE1, and DE2.

### Tissue collection and processing

After 120 days of experimentation, animals were weighed, intraperitoneally anesthetized with thiopental (40 mg/kg body weigh; Abbott Laboratories, Chicago, IL, USA), and sacrificed. Blood was collected by heart puncture and stored in heparinized flasks, and the plasma portion was used to evaluate the levels of glycemia and glycated hemoglobin [29].

After laparotomy, the large intestine was collected, and its length and circumference were measured to obtain the area. Additionally, segments of the proximal colon were collected. Some samples were designated to the histological study of the intestinal wall and the Periodic Acid-Schiff histochemical technique to identify goblet cells. The other samples were subjected to whole-mount preparations stained using the Giemsa method for the morphoquantitative study of total myenteric population.

### Study of the intestinal wall

Samples of the proximal colon were opened along the mesocolic region and distended on an isoprene plate. The mucosa was then exposed and washed with saline solution to remove feces. Segments were then fixed in Bouin solution for 12 h and stored in 70% alcohol. Segments underwent two mean inclusions: (1) in paraffin to obtain semi-seriated sections of 7 μm thickness each and subjected to hematoxilin and eosin (HE) staining and (2) in historesin (LKB, Technovit 7100) to obtain semi-seriated sections of 7 μm thickness each to histochemically identify glycoproteins present in intestinal goblet cells using the PAS method.

Images of the obtained sections were captured using a high-resolution camera (Q Color 3, Olympus America) coupled to a light microscope (Olympus BX40). Morphometric and quantitative analyses were performed with the aid of Image Pro Plus^® ^4.5 image analysis software (Media Cybernetics, Silver Spring, MD, USA).

Images of the sections stained with HE were used to measure the thickness of the mucous and muscular tunic and total intestinal wall at 10 points chosen randomly, with five sections per animal, for a total of 250 measures per group. Images of sections obtained using the PAS histochemical method were used to quantify goblet cells present in 50 images per animal (40× objective). The area of each image was 0.01 mm^2^. Results are expressed as the number of goblet cells per mm^2^.

### Quantitative and morphometric study of myenteric neurons

Proximal colon samples from five animals per group were collected to evaluate the total population of myenteric neurons using the Giemsa method [30]. After collection, segments were washed in saline solution, filled with Giemsa fixative, and embedded in this solution for 24 h. Segments were opened at the mesocolic region and microdissected under a stereomicroscope with transillumination by removing the mucosa and submucosa layers and preserving the muscular tunic and serosa. Sequential whole-mount preparations were stained by the Giemsa method in Sorensen's phosphate buffer 0.1 N (pH 6.9) for 24 h at room temperature. Sections were then dehydrated in a series of increasing ethanol concentrations, diaphanized in xylol, and mounted and coverslipped on slides using Permount^®^.

#### Quantitative analysis

**Figure 1:**
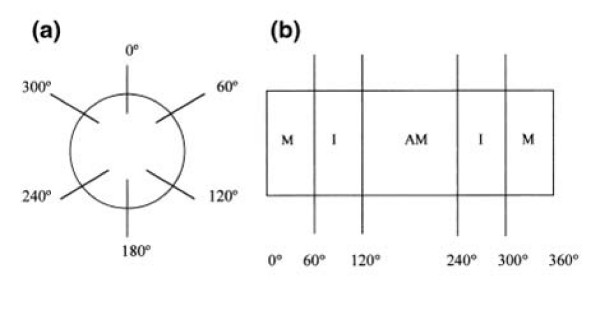
**(a) Transverse section of the intestine: 0° represents the mesocolon insertion, 0°-60° and 300°-360° the mesocolic regions, 60°-120° and 240°-300° the intermediate region, and 120°-240° the antimesocolic region of the intestinal circumference**. (b) Intestinal segment opened along the mesocolic edge (M, mesocolic region; I, intermediate region; AM antimesocolic region) [31].

Using an Olympus optical microscope with a 40× objective, we quantified the number of myenteric neurons present in 40 microscopic fields in the intermediate regions (60°-120°, 240°-300°) and 40 microscopic fields in the anti-mesocolic regions (120°-240°) of the intestinal circumference, with 0° as the mesocolon insertion (Figure 1) [31]. Each field corresponded to 0.18 mm^2^, for a total of 14.4 mm^2^/animal.

#### Morphometric analyses

To evaluate the area of the cellular bodies (cellular profile) of myenteric neurons, 500 neurons per group were measured. Images were obtained using a high-resolution camera coupled to an optical microscope. Morphometric analyses were performed using Image Pro Plus^® ^4.5 image analysis software (Media Cybernetics, Silver Spring, MD, USA).

### Statistical analysis

Statistical analysis was performed using GraphPad Prism^® ^3.0 (GraphPad Software, San Diego, CA, USA). Analysis of variance (ANOVA) was followed by Tukey's *post hoc *to compare mean values. Levels of *p *< 0.05 were considered statistically significant. Results are expressed as mean ± standard error.

## Results

Streptozotocin promoted the development of a diabetic syndrome in animals from groups D, DE1, and DE2, verified as an increase in glycemia and glycated hemoglobin (Table 1). During the experimental period, typical clinic manifestations of the disease were observed, such as polyphagia, polydipsia, polyuria, and a reduction in body weight compared with normoglycemic animals.

**Table 1:** Glycemia, glycated hemoglobin, final body weight, and large intestine area.

Group	Glycemia (mg/dl)	Glycated hemoglobin (%)	Final weight (g)	Intestinal area (cm^**2**^)
N	178.2 ± 17.3^a^	3.16 ± 0.13^a^	450.3 ± 15.6^a^	18.75 ± 1.6^a^
NE1	147.2 ± 8.3^a^	3.64 ± 0.26^a^	419.2 ± 12.6^a^	20.35 ± 59^a^
NE2	128 ± 6.4^a^	2.54 ± 0.05^b^	406.7 ± 9.8^a^	20.42 ± 3.7^a^
D	489.6 ± 7.2^b^	6.15 ± 0.45^c^	268.2 ± 14.5^b^	36.82 ± 3.8^b^
DE1	525.2 ± 23.2^b^	6.50 ± 0.10^c^	291.3 ± 21.9^b^	32.44 ± 5.2^b^
DE2	319.7 ± 3.6^c^	4.78 ± 0.13^d^	290 ± 21.7^b^	29.46 ± 3.7^b^

N, normoglycemic; NE1, normoglycemic supplemented with vitamin E (0.1%); NE2, normoglycemic supplemented with vitamin E (2%); D, diabetic; DE1, diabetic supplemented with vitamin E (0.1%); DE2, diabetic supplemented with vitamin E (2%). Mean ± standard error (*n *= 5). Means followed by different letters in the same column are statistically different (Tukey's test, *p *< 0.05).

Supplementation with vitamin E (2%) significantly reduced glycemia levels and glycated hemoglobin compared with animals from groups D and DE1 (*p *< 0.001). This vitamin E concentration also reduced glycated hemoglobin in the normoglycemic supplementation group (NE2). Supplementation with vitamin E 0.1% did not alter glycemia levels or glycated hemoglobin in group DE1 compared with group D (*p *< 0.05). Animals from groups DE1 and DE2 exhibited less weight loss compared with untreated diabetic animals (*p *> 0.05) (Table 1).

The area of the large intestine was significantly greater in diabetic animals compared with animals from group N (*p *< 0.001) and not altered by either concentration of vitamin E supplementation (Table 1).

### Morphometric analysis of the intestinal wall

**Figure 2:**
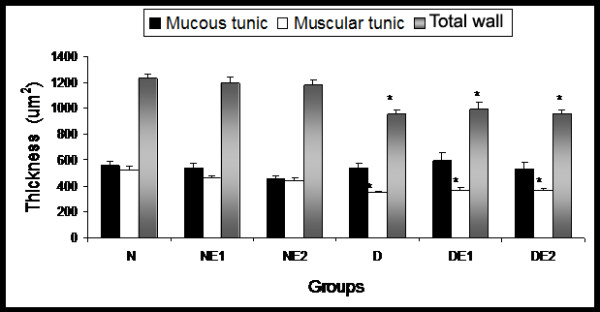
**Morphometry of the intestinal wall, mucous tunic, muscular tunic, and total wall of rats from groups N (normoglycemic), NE1 (normoglycemic supplemented with vitamin E 0.1%), NE2 (normoglycemic supplemented with vitamin E 2%), D (diabetic), DE1 (diabetic supplemented with vitamin E 0.1%), and DE2 (diabetic supplemented with vitamin E 2%)**. Mean ± standard error (*n *= 5). *p < 0.05, compared with the other groups.

The total intestinal wall and muscular tunic in diabetic animals were significantly reduced compared with non-diabetic animals (*p *< 0.05) (Figure 2) but maintained their typical histological organization. Vitamin E supplementation did not promote any reversal of this parameter. The thickness of the mucous tunic was maintained among groups.

### Quantitative analysis of intestinal goblet cells

The number of goblet cells present in 0.5 mm^2 ^per animal was not statistically different among groups. The average number of goblet cells for each group was: N (8896 ± 400.1), NE1 (7613 ± 344.5), NE2 (8246 ± 543.7), D (8532 ± 255.4), DE1 (7427 ± 392.5), and DE2 (7315 ± 376.3).

### Quantitative analysis of total myenteric neuron population

Considering the whole-mount preparations stained by the Giemsa method, observing the maintenance of ganglia arrangement was possible, which ranged according to number and size, with sparse isolated neurons independent of group.

**Figure 3:**
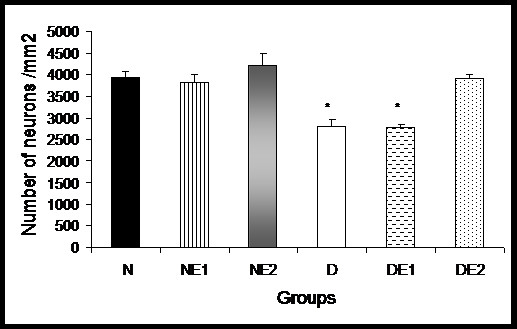
**Number of myenteric neurons quantified in 14.4 mm^2 ^of the proximal colon of animals from groups N (normoglycemic), NE1 (normoglycemic supplemented with vitamin E 0.1%), NE2 (normoglycemic supplemented with vitamin E 2%), D (diabetic), DE1 (diabetic supplemented with vitamin E 0.1%), and DE2 (diabetic supplemented with vitamin E 2%)**. Mean ± standard error (*n *= 5). *p < 0.05, compared with the other groups.

Neuronal counting was performed in the antimesocolic and intermediate regions of the proximal colon. These values were not significantly different; therefore, these data were collapsed. The experimental diabetes condition promoted a reduction in the number of myenteric neurons in groups D (2805 ± 176.9) and DE1 (2797 ± 70.0) compared with groups N (3929 ± 149.7), NE1 (3820 ± 179.0), and NE2 (4224 ± 262.8) (*p *< 0.05). A positive effect of vitamin E (2%) was confirmed. Neuronal population in group DE2 (3923 ± 78.95) was significantly different compared with groups D and DE1 (*p *< 0.05) (Figure 3).

### Morphometric analysis of total myenteric neuron population

**Figure 4:**
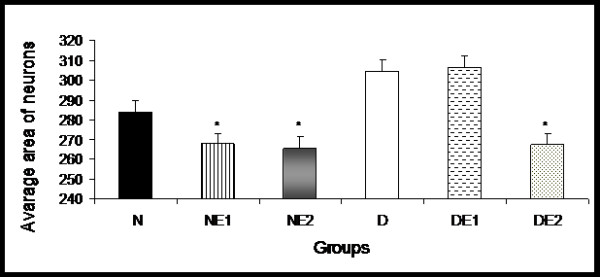
**Average areas of myenteric neurons in the proximal colon from groups N (normoglycemic), NE1 (normoglycemic supplemented with vitamin E 0.1%), NE2 (normoglycemic supplemented with vitamin E 2%), D (diabetic), DE1 (diabetic supplemented with vitamin E 0.1%), and DE2 (diabetic supplemented with vitamin E 2%)**. Mean ± standard error (*n *= 5). *p < 0.05, compared with groups D and DE1.

The mean areas for myenteric neurons (μm^2^) in the studied groups were the following: N (283.9 ± 16.3), NE1 (267.7 ± 13.5), NE2 (265.6 ± 30.4), D (304.4 ± 13.7), DE1 (306.5 ± 13.3), and DE2 (267.3 ± 13.4). Comparisons between groups N and D did not reveal any significant differences (*p *> 0.05) (Figure 4). Despite the lack of differences, animals from group DE2 exhibited a reduction in cellular body area compared with groups D and DE1 (*p *< 0.05). A range from 201 to 300 μm^2 ^was the predominant cellular profile for all groups.

## Discussion

Streptozotocin effectively induced experimental diabetes in the present study, reflected by an increase in glycemia and glycated hemoglobin in animals from groups D, DE1, and DE2. The typical characteristics of a diabetic condition were also observed when these animals were compared with groups N, NE1, and NE2, such as polyphagia, polydipsia, and polyuria. These typical clinical manifestations were not experimentally assessed in the present experiment but were generally observed during the animal's daily behavior. These manifestations in diabetic rodents have been frequently found in our previous experiments which induced experimental diabetes with the same drug [14,32].

Diabetic animals that received vitamin E (2%) supplementation exhibited a significant reduction in glycemia and glycated hemoglobin compared groups D and DE1, indicating a positive effect of this antioxidant on blood glycemia. Shirpoor et al. [22], in a study performed with diabetic rats that received vitamin E (300 mg/kg body weight), observed a significant reduction in blood glucose and glycated hemoglobin in treated diabetic animals compared with untreated diabetic animals. Another study also performed with diabetic rats supplemented with vitamin E (650 mg/kg body weigh) demonstrated that glucose blood levels were reduced in supplemented diabetic animals compared with untreated diabetic animals [33]. Although these authors observed a reduction in glycemia with lower concentrations of vitamin E than those used in the present study, daily intake of vitamin E (2%) in chow averaged 900 mg. The route of administration was also different (vitamin E in water [22] and vitamin E through gavage [34]), suggesting that the route of administration is important when the effects of this antioxidant are evaluated and that supplementation in chow demands higher vitamin E concentrations [23].

The mechanism by which the antioxidant reduced blood glucose levels has not yet been established. Antioxidants increase glucose metabolism in peripheral tissues, leading to a decrease in plasma glucose levels [33]. This fact could explain the reduction in glycated hemoglobin promoted by vitamin E in animals from group NE2. Further studies are needed to determine the relationship between vitamin E dose and response and to establish the mechanism by which this antioxidant reduces blood glucose levels in experimental diabetes.

The area of the large intestine in diabetic animals was significantly larger. A dimensional increase in organs in diabetic animals is often reported in the literature, for both the small intestine [3,17,33,35] and large intestine [13,36]. Larger cecum dimensions were observed by Zanoni et al. [13], who attributed this change to a decrease in muscular fiber tone, thus leading to greater dilatation. In contrast, Miranda Neto et al. [36] attributed the larger dimension found in the distal colon of diabetic rats as an effect of the nitrergic population (inhibitory neurons), thus leading to a decrease in muscular tone. In the present study, the increase in the large intestine may be attributable to several related factors, including a reduction in intrinsic innervation in this organ, reflected by decreased myenteric neuronal population and decreased thickness of the muscular tunic associated with intestinal motility.

Vitamin E supplementation did not induce any effects on the dimensions of the large intestine in the studied groups. Shirpoor et al. [37] observed that in the small intestine of diabetic rats receiving vitamin E (300 mg/kg body weight) intestinal length was not altered.

The thickness of the total intestinal wall and muscular tunic was reduced in diabetic animals. Data from the literature reveal that the small intestine of rats responds to experimental diabetes by increasing the total wall thickness and thickness of the muscular tunic [3]. These results do not necessarily contradict with the present study because the previous study maintained an acute diabetes condition and our present model maintained a chronic condition. The decrease in total intestinal wall thickness is a reflex to a reduction in the muscular tunic, which is a consequence of innervation promoted by a loss of myenteric neurons.

The thickness of the mucous tunic did not exhibit any significant alterations in any of the studied groups. This result is corroborated by a maintenance of crypts in the proximal colon of rats with chronic diabetes [32], possibly attributable to physiological adaptations in which an increase in proliferation of intestinal crypts is not observed. Other studies reported hypertrophy [3] and hyperplasia [7] in the mucosa of the small intestine in rats subjected to an acute experimental diabetes model. The results obtained with the mucous, muscular tunic, and total wall suggest that variations in these parameters are directly related to the period of maintenance of the diabetic state. Another factor suggested by Belai et al. [33] is that diabetes mellitus differentially affects the intestines, with the small intestine more susceptible than the large intestine.

The thickness of the total intestinal wall, mucous, and muscular tunic of the proximal colon in diabetic animals was not affected by vitamin E supplementation, independent of concentration. An absence of effect of vitamin E was observed by Shirpoor et al. [37] for villi height and crypt depth in the small intestine of diabetics animals. Glutamine supplementation, a precursor of an important antioxidant agent, glutathione, also did not alter crypt depth or cell proliferation in the colonic mucosa of diabetic rats [32].

Quantitative analysis of goblet cells did not reveal significant differences among groups in a study by Mantle et al. [5], in which an increase in number of goblet cells in intestinal villi was found in the small intestine of rats with acute diabetes. An increase in the number of goblet cells in crypts of the small intestine of diabetic mice was reported by Ettarh and Carr [38], who suggested that the increase in goblet cells directly related to an increase in mucous tunic. These results suggest the conservation of the mucous tunic in diabetic animals, which would justify the maintenance of this cellular population as a morphofunctional adaptation to the chronic diabetes condition.

Neuronal quantification revealed a significant reduction in the number of myenteric neurons in animals from the diabetic group compared with normoglycemic animals. This is an expected result for rats [9,10,12-14], supported by histochemical and immunohistochemical data from different segments of the gastrointestinal tract [11,32,39]. The marked glycemia caused by the diabetic condition has been considered to be the main factor involved in the development and progression of neuropathy, promoting abnormalities in blood flow and increasing vascular permeability, in addition to decreasing neuronal cell trophic factors [16]. A reduction in antioxidant systems in diabetes, including vitamins A, C, and E, glutathione, and glutathione peroxidase, also contributes to the development of neuropathy [40].

Administration of vitamin E (2%) had a neuroprotective effect, conserving the number of myenteric neurons in group DE2 compared with groups D and DE1. Cotter et al. [23] found that the reduction in the velocity of nervous system conductance could be attenuated in diabetics rats with vitamin E at a concentration of 0.1%. According to these authors, in experimental diabetes, glycemic levels and the increase in free radicals are excessive compared with patients whose diabetes is well controlled. This may explain why a neuroprotective effect was not observed in group DE1 (0.1%). The decreased neuronal loss in group DE2 could be attributed to the fact that vitamin E potentiates the antioxidant system, in which erythrocytes in diabetic rats promote an increase in glutathione and antioxidant enzymes, such as superoxide dismutase and glutathione peroxidase [18]. Additionally, the development of diabetic neuropathy is associated with mitochondrial dysfunction which consequently generates reactive oxygen species, which are related to activation of a cascade of events leading to apoptosis [41,42]. Therefore, vitamin E supplementation may have minimized these events to reduce neuronal death in group DE2.

When the average area of cellular bodies was assessed, significant differences were not observed when diabetic groups were compared with normoglycemic animals. The increase in the area of neuronal cell bodies was observed in diabetic rats [8,39], an effect attributed to an increase in neuronal synthesis compensating for the neuronal death, thus leading to an increase in cellular volume [39].

The results of the present study are similar to Tashima et al. [32] with regard to the area of myosin-V neurons in the proximal colon of diabetic rats. The myosin-V immunohistochemistry technique is a marker for total neuronal population. However, Zanoni et al. [39] found an increased area of these VIPergic neurons in diabetics rats, possibly attributable to greater neuropeptide synthesis by these cells. According to Tashima et al. [32], diabetes mellitus directly affects neuronal subpopulations in the gastrointestinal tract, suggesting that techniques which identify total neuronal population, such as the myosin-V immunohistochemistry and Giemsa techniques, do not demonstrate these alterations.

Importantly, a statistically significant decrease in the area of neuronal cellular bodies was observed in group DE2 compared with groups D and DE1, indicating a positive influence of vitamin E (2%) on this parameter. These results are corroborated by Zanoni et al. [39], in which supplementation with ascorbic acid prevented the increase in VIPergic neurons in the ileum of diabetic rats. Our results indicate that vitamin E (2%) has a neurotrophic role by maintaining neuronal cellular bodies in diabetic animals.

## Conclusion

Supplementation with vitamin E (2%) in rats with chronic diabetes mellitus positively influenced glycemia, had neuroprotective effects on myenteric neurons, and did not affect the morphological characteristics of the intestinal wall.

## Competing interests

The authors declare that they have no competing interests.

## Authors' contributions

LPR designed the study, collected the data, analyzed the statistics, and wrote the manuscript. MRMN and JNZ helped JFL manage the data and critically reviewed the manuscript. RVFP, EAT, GVR, and CRS critically reviewed the manuscript and contributed to the study design. All authors read and approved the final version of the manuscript.

## Pre-publication history

The pre-publication history for this paper can be accessed here:

http://www.biomedcentral.com/1471-230X/9/88/prepub
